# Super-resolution T2-weighted 4D MRI for image guided radiotherapy

**DOI:** 10.1016/j.radonc.2018.05.015

**Published:** 2018-12

**Authors:** Joshua N. Freedman, David J. Collins, Oliver J. Gurney-Champion, Jamie R. McClelland, Simeon Nill, Uwe Oelfke, Martin O. Leach, Andreas Wetscherek

**Affiliations:** a Joint Department of Physics, The Institute of Cancer Research and The Royal Marsden NHS Foundation Trust, London, UK; b CR UK Cancer Imaging Centre, The Institute of Cancer Research and The Royal Marsden NHS Foundation Trust, London, UK; c Centre for Medical Image Computing, Department of Medical Physics and Biomedical Engineering, University College London, UK

**Keywords:** 4D MRI, T2w 4D MRI, Super resolution, Motion vector field, Radiotherapy treatment planning

## Abstract

**Background and purpose:**

The superior soft-tissue contrast of 4D-T2w MRI motivates its use for delineation in radiotherapy treatment planning. We address current limitations of slice-selective implementations, including thick slices and artefacts originating from data incompleteness and variable breathing.

**Materials and methods:**

A method was developed to calculate midposition and 4D-T2w images of the whole thorax from continuously acquired axial and sagittal 2D-T2w MRI (1.5 × 1.5 × 5.0 mm^3^). The method employed image-derived respiratory surrogates, deformable image registration and super-resolution reconstruction. Volunteer imaging and a respiratory motion phantom were used for validation. The minimum number of dynamic acquisitions needed to calculate a representative midposition image was investigated by retrospectively subsampling the data (10–30 dynamic acquisitions).

**Results:**

Super-resolution 4D-T2w MRI (1.0 × 1.0 × 1.0 mm^3^, 8 respiratory phases) did not suffer from data incompleteness and exhibited reduced stitching artefacts compared to sorted multi-slice MRI. Experiments using a respiratory motion phantom and colour-intensity projection images demonstrated a minor underestimation of the motion range. Midposition diaphragm differences in retrospectively subsampled acquisitions were <1.1 mm compared to the full dataset. 10 dynamic acquisitions were found sufficient to generate midposition MRI.

**Conclusions:**

A motion-modelling and super-resolution method was developed to calculate high quality 4D/midposition T2w MRI from orthogonal 2D-T2w MRI.

Magnetic resonance image guided radiation therapy (MRIgRT) is an emerging treatment approach that takes advantage of the exquisite soft tissue contrast [1], motion resolving capabilities and functional information offered by MRI, to improve treatment delivery in radiotherapy [2], [3]. Four-dimensional (4D) T2-weighted (T2w) MRI is a promising candidate for use in radiotherapy treatment planning (RTP) of moving anatomy [4], [5], [6]. For instance, a mid-position (time-weighted mean position of the respiratory cycle) (MidP) image can be calculated from 4D-T2w MRI and co-registered with MidP-CT to facilitate target delineation [7], [8]. 4D-T2w MRI might also be repeatedly acquired between fractions and used to update treatment plans. In a hybrid MRIgRT treatment setting [2], [9], [10], [11], [12], MidP/4D-T2w MRI has potential for positioning and beam-on guidance [13], [14], [15].

Previously, 4D-T2w MRI has been generated by sorting dynamically acquired two-dimensional (2D) T2w MRI both prospectively and retrospectively with the aid of an image-driven or external respiratory signal [16], [17], [18], [19], [20], [21], [22], [23], [24], [25]. In retrospective methods, 2D-T2w MRI is continuously acquired and then sorted into respiratory-bins. Unlike external respiratory signals, such as a respiratory bellows [17], image-driven respiratory signals (IRS) are available from the acquired data and do not disturb the measurement process [19]. However, IRS exhibit a phase-shift [26] between different slices, since they are calculated on a slice-by-slice basis.

Retrospective binning methods suffer from missing-data artefacts, when slices are not acquired for particular respiratory phases [21]. Furthermore, a 2D distortion correction is usually applied, which is suboptimal, because 3D geometrical fidelity is essential for RTP [27]. An additional problem are highly anisotropic voxel sizes, due to the large slice-thickness required to obtain a sufficient field-of-view (FoV) and signal-to-noise ratio [17], [21]. Staircase (stitching) artefacts, due to both the large slice-thickness and respiratory variations, are observed when reformatting data into orthogonal views [16]. Slice-thickness can potentially be reduced with a super-resolution reconstruction [28], which combines several low-resolution images containing independent information into one super-resolution image. The concept of super-resolution reconstruction has been applied in 4D-MRI [29], [30], [31], but has not yet been translated to 4D-T2w MRI.

In this article, an automated binning and super-resolution reconstruction method is introduced to calculate high-resolution distortion corrected MidP/4D-T2w MRI, without missing-data artefacts, from dynamically acquired axial and sagittal 2D-T2w MRI. The performance of 4D-T2w MRI is assessed in phantom experiments and compared to low-resolution 2D-T2w MRI using colour-intensity projection images (CIPs) [32], [33]. The influence of the acquired number of dynamic acquisitions on the MidP-T2w MRI quality was investigated by retrospectively subsampling the data-set.

## Materials and methods

### Data acquisition

Eight healthy volunteers (aged 24–35, 4 female) were included in this study after giving written informed consent. Volunteers were scanned in both sagittal and axial orientations, using 30 repeated dynamic acquisitions (referred to below as dynamics), with a 2D-T2w half Fourier turbo spin echo (HASTE) sequence (voxel-size 1.5 × 1.5 × 5 mm^3^, echo time 64 ms, effective repetition time per slice 13.6–14.2 s, in-plane FoV 264 × 384 mm^2^, 40–60 slices that were scanned interleaved, readout bandwidth 590 Hz/Px, refocusing flip-angle 90–92°, total acquisition time 15.0–18.4 min) in free breathing at 1.5 T (MAGNETOM Aera; Siemens Healthcare, Erlangen, Germany). An in-house developed coil holder was used to prevent compression of the thorax by the 18-channel receive array. Scans were acquired with the patient’s arms adjacent to the thorax. The default gradient non-linearity 2D distortion correction was disabled.

### Workflow

**Fig. 1:**
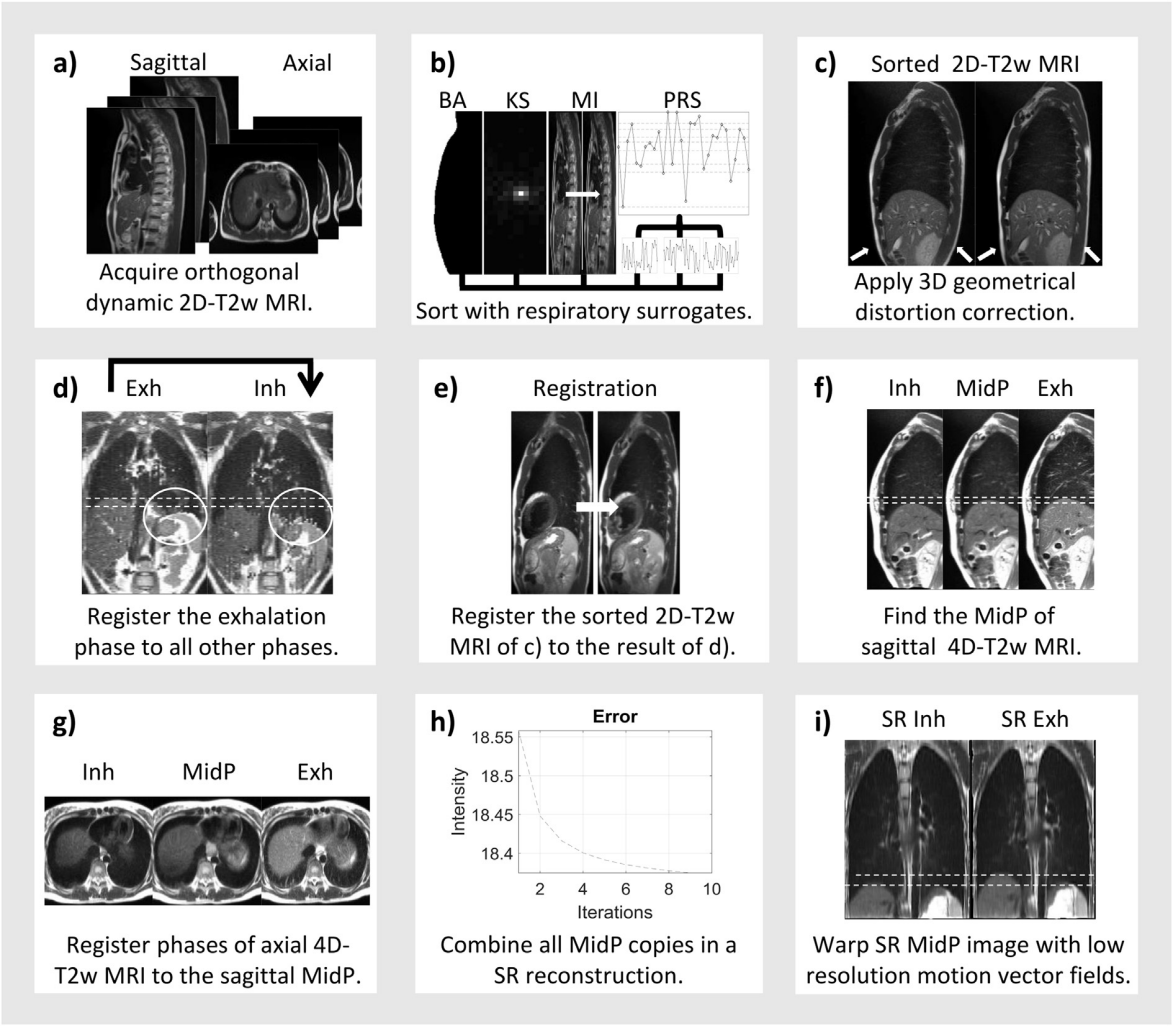
(a) Axial and sagittal 2D-T2w MRI are continuously acquired, (b) retrospectively sorted by the principle respiratory surrogate (PRS), which combines body area (BA), artificial k-space (KS) and mutual information (MI), and (c) distortion corrected. (d) For both axial and sagittal 4D-T2w MRI, stitching and binning artefacts, visible within the white circle, are reduced by registering the exhalation phase to all other respiratory phases. (e) Sorted images in (c) are corrected by registering to the results of (d). (f) Each phase of the sagittal 4D-T2w image of (e) is warped to midposition (MidP). (g) Each phase of the axial 4D-T2w image in (e) is registered to the sagittal MidP. (h) Super-resolution (SR) reconstruction is performed using the sagittal and axial MidP copies. (i) SR 4D-T2w MRI is obtained by warping the SR MidP image with the low-resolution motion vector fields calculated from the corrected sagittal 4D-T2w MRI in (f).

Low-resolution sagittal and axial 4D-T2w MRI were first calculated with a binning and motion-modelling method and then aggregated into a high-resolution MidP-T2w image using a super-resolution reconstruction as described below. High-resolution 4D-T2w MRI was then calculated by transforming the MidP-T2w image with motion vector fields calculated from the low-resolution sagittal 4D-T2w MRI. A summary is given in Fig. 1. All calculations were carried out in MATLAB (version 2017a; The MathWorks, Natick, MA) on an Intel Xeon E5-1660 processor with 8 cores at 3 GHz and 64 GB of memory.

The following binning and motion-modelling workflow was separately undertaken for both the axial and sagittal acquisitions.

### Binning

For each slice, five IRS were calculated using the concepts of: body area [20], mutual information [23], and artificial k-space [19]. A binary mask (background and lungs = 0, remaining = 1) was calculated by thresholding each acquired image; thresholds were automatically calculated as 2/5th of the mean image intensity. Body area was identified as the largest connected component. Mutual information was calculated between exhalation and all other phases, where exhalation was determined as the minimum of the pre-calculated body area IRS. Three k-space IRS were generated from the magnitude of the centre (0,0), centre-upper (0,1) and centre-right (1,0) pixels of the 2D Fourier transform of each magnitude image [19]. Employing a partial Fourier acquisition did not affect calculation of k-space IRS from the image data, because only the magnitude signal of the artificial k-space data points was used. All five IRS were combined into one principle respiratory signal (PRS) using principle component analysis (first principle component).

The PRS was employed to retrospectively sort each slice into eight respiratory-bins. The edges of the respiratory-bins were defined using percentiles of the PRS amplitude values, such that there was an equal amount of data in each respiratory-bin. Magnitude images assigned to the same respiratory-bin were averaged. Defining respiratory-bins separately for each slice mitigates missing-data artefacts, as all bins can be filled [21], but introduces stitching artefacts or phase-shifts between slices [26], due to the particular sampling scheme or changes in the respiratory pattern during measurement. Finally a 3D gradient non-linearity distortion correction was applied to each phase of the sorted images, using the spherical harmonic coefficients provided by the vendor [34].

### Motion-modelling

Compared to other respiratory phases, exhalation exhibited reduced stitching and binning artefacts. We used the exhalation image to correct the artefacts exhibited in the remaining respiratory phases.

In this approach, the exhalation image was registered to all eight respiratory phases using a diffeomorphic Demons 3D non-rigid registration [35] with diffusion-like smoothing (*σ* = 1.0 pixel), which greatly increased robustness to stitching artefacts. The *σ* parameter was optimised heuristically in a subset of the volunteers by visually assessing the quality of the registered images. The value *σ* = 1.0 pixel was found to provide the best compromise between reduction of stitching artefacts and over-regularisation of respiratory motion. The calculated motion vector fields were then applied to warp the exhalation image to all other respiratory phases; resulting in a simulated 4D-T2w MRI with reduced stitching artefacts. Afterwards, each slice from the original sorted 4D-T2w MRI was corrected by registering to the corresponding slice and phase in the simulated 4D-T2w MRI, using a similar Demons 2D non-rigid registration (*σ* = 1.0 pixel). This approach retained the independent information needed for the super-resolution reconstruction.

### Super-resolution reconstruction

Using NiftyReg [36], [37], eight copies of the MidP image [7] were generated from all eight phases of the sagittal 4D-T2w image. NiftyReg was employed to generate eight additional copies by non-rigidly registering all eight respiratory phases of the axial 4D-T2w image to the sagittal MidP copy that was generated from the corresponding respiratory phase. All independent MidP copies were interpolated to isotropic resolution (1.0 × 1.0 × 1.0 mm^3^) and averaged to form an initial guess [28] for the super-resolution reconstruction [38]. High-resolution MidP-T2w MRI was calculated by iteratively back-projecting the differences between the low-resolution MidP copies and the super-resolution image that was convolved with a non-isotropic Gaussian point spread function [39].

Motion vector fields, which were calculated by non-rigidly registering the respiratory phases of low-resolution sagittal 4D-T2w MRI, were applied to high-resolution MidP-T2w MRI to obtain super-resolution 4D-T2w MRI. Because super-resolution reconstruction was limited to the overlapping FoV of the sagittal and axial images, the original low-resolution axial images were used outside the thorax (arms). A detailed overview of the super-resolution reconstruction workflow can be found in the Supplemental Material.

### Verification: image resolution

Deformable image registration and interpolation steps in the workflow result in smoothing of the image data and potentially a loss of resolution. An edge-detection method was used to compare the resolutions of the unprocessed ground-truth, initial guess and super-resolution reconstructed images. In this approach a 2D region of interest (ROI) was manually defined on the anterior skin-air boundary in the central axial slice of each volunteer. A Gaussian function was then fitted to the derivative of each measured line of intensity $y^{\prime }(x)$ (matrix of size [1, *N*]) in the ROI.

$$y_{0}=\underset{a,\mu ,\sigma }{\text{argmin}}\overset{N}{\underset{x=1}{\sum }}{\left(y^{\prime }(x)-a\;\text{exp}\left[-\frac{1}{2}{\left(\frac{\text{x}-\mu }{\text{b}}\right)}^{2}\right]\right)}^{2}$$

Here, $y_{0}$ describes the result of fitting the Gaussian function of height a, position μ, and width b to the derivative $y^{\prime }(x)$ using least squares minimisation. The image resolution was obtained by averaging the calculated width b over all lines in the ROI. For each volunteer, the same 2D ROI was used to measure the image resolution on the unprocessed ground-truth, initial guess and super-resolution reconstructed images.

### Verification: phantom measurements

The QUASAR^TM^ MRI-compatible Respiratory Motion Phantom (Quasar, Modus Medical Devices Inc., London, Canada), into which a kiwi and corn test-object was inserted, was employed to validate the binning and motion-modelling workflow. Two sinusoidal waveforms (S1-S2) with period and amplitude: (4 s, 15 mm) and (3 s, 20 mm), and two randomly chosen volunteer waveforms (S3-S4) (self-gating signal of a radial T1 weighted sequence that was acquired in the same session as T2w MRI [40]) with amplitude 15 mm were chosen to drive the phantom. Translational motion of the phantom insert along the bore was coupled to rotation around the axis of translational motion. The rotation angle θ was given by the position A (between −20 and 20 mm):

$$\theta (A)=\sin^{-1}\left(\frac{1}{2}\sqrt{1-{\left(\frac{A}{20}\right)}^{2}}\right)$$

The volunteer waveform amplitude values of 15 mm were chosen to be representative of patient respiratory tumour motion [41]. Images were obtained with identical sequence parameters to the volunteer acquisitions, except that they were acquired in the coronal and sagittal orientations. Because the main compartment of the phantom was a static water tank and only the insert exhibited motion, the PRS of body area and artificial k-space were not applicable and the position of the lower kiwi boundary, which was measured using an edge-detection method [5], was used instead. Furthermore, the Demons non-rigid registration was optimised (*σ* = 0.5 pixel) to capture the rotation of the test-object. Using the same edge-detection method, the MidP displacement was compared to the mean displacement over *N* dynamics (*N* = 10, 15, 20, 25 and 30) measured in ground-truth low-resolution images.

### Verification: colour-intensity projections

CIPs can be used to encode the intensity variation within a 4D image set by colour, and for objects similar to an isolated lung tumour, colour approximately represents the time spent by the object in any one position [32], [33]. CIPs display grayscale values when intensity does not change at a particular position in the 4D image set. Calculated CIPs are independent of the binning and motion-modelling workflow and therefore enable a comparison of the motion information exhibited by low-resolution 4D-T2w MRI and the unprocessed ground-truth phantom data. For the volunteer images, CIPs were calculated from both the unprocessed ground-truth data and the generated super-resolution 4D-T2w MRI.

### MidP dependence on number of dynamics

To minimise acquisition time, the number of dynamic images required to obtain a representative MidP image was investigated by retrospectively discarding data prior to generating the 4D-MRI. Following Fig. 1(b–f), MidP images were calculated from only the first 10–30 sagittal dynamics. For each MidP image, the position of the right hemi-diaphragm was calculated for five consecutive sagittal slices, using an edge-detection method [5], and then averaged. It was compared to the diaphragm position of the MidP image obtained with 30 dynamics, which was considered to contain sufficient dynamics for data completeness based on [21]. In order to separate the impact of the number of dynamics *N* from possible changes in respiratory pattern during acquisition, the change in MidP was estimated from the unprocessed ground-truth data, by calculating the average diaphragm position over *N* dynamics for *N* = 10–30; carried out using the edge-detection method on the same slices and settings as for the above MidP images.

## Results

**Fig. 2:**
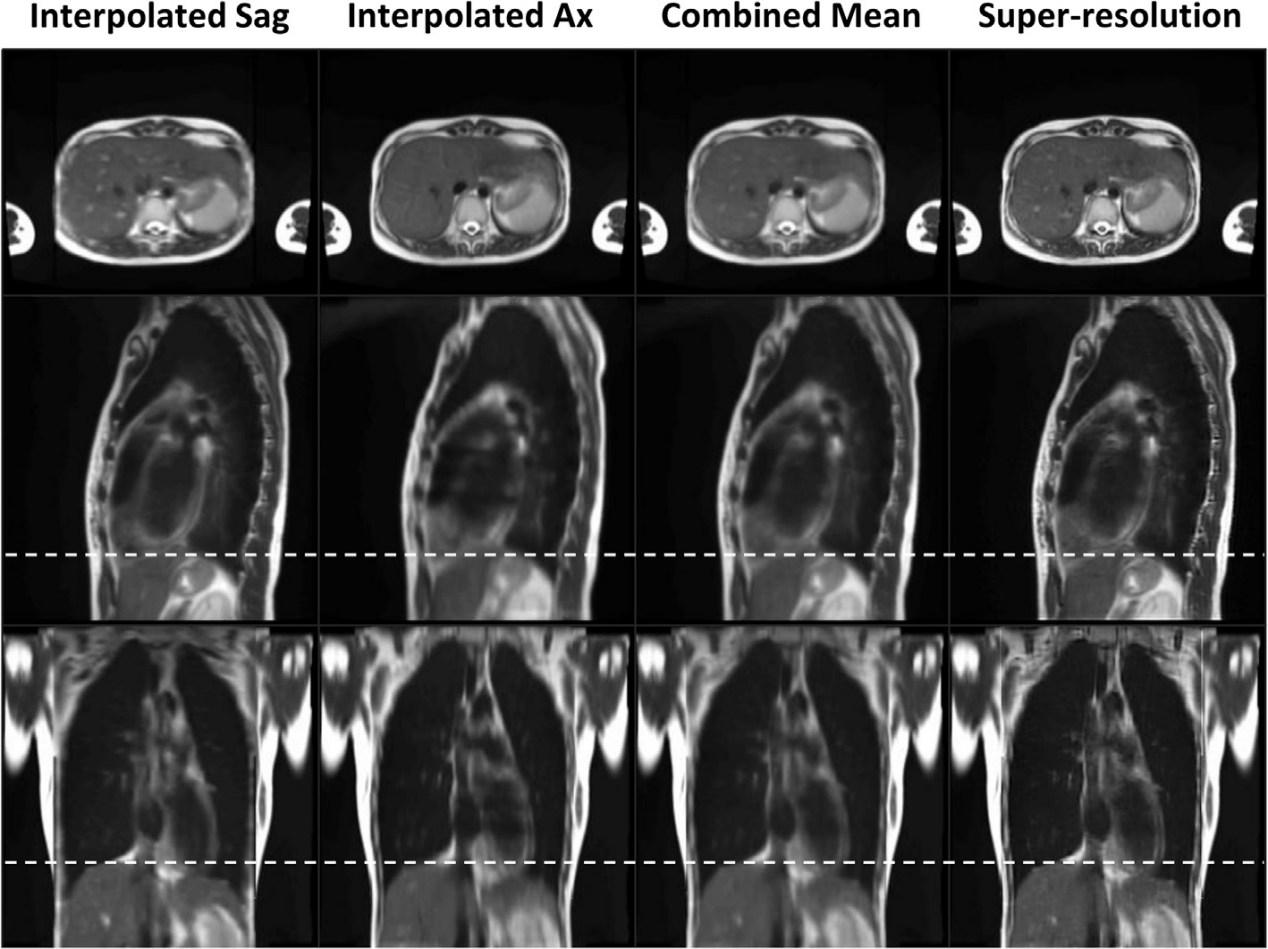
A comparison between the combined and individual mean interpolated sagittal (Sag) and axial (Ax) images, and the super-resolution reconstruction. Dashed white lines aid evaluation of the relative liver dome positions in the different midposition (of the respiratory cycle) images. The suppressed average cardiovascular pulsations in the Interpolated Sag and Ax columns (e.g. bright liver vessels in the axial view) were retained in the super-resolution image.

**Fig. 3:**
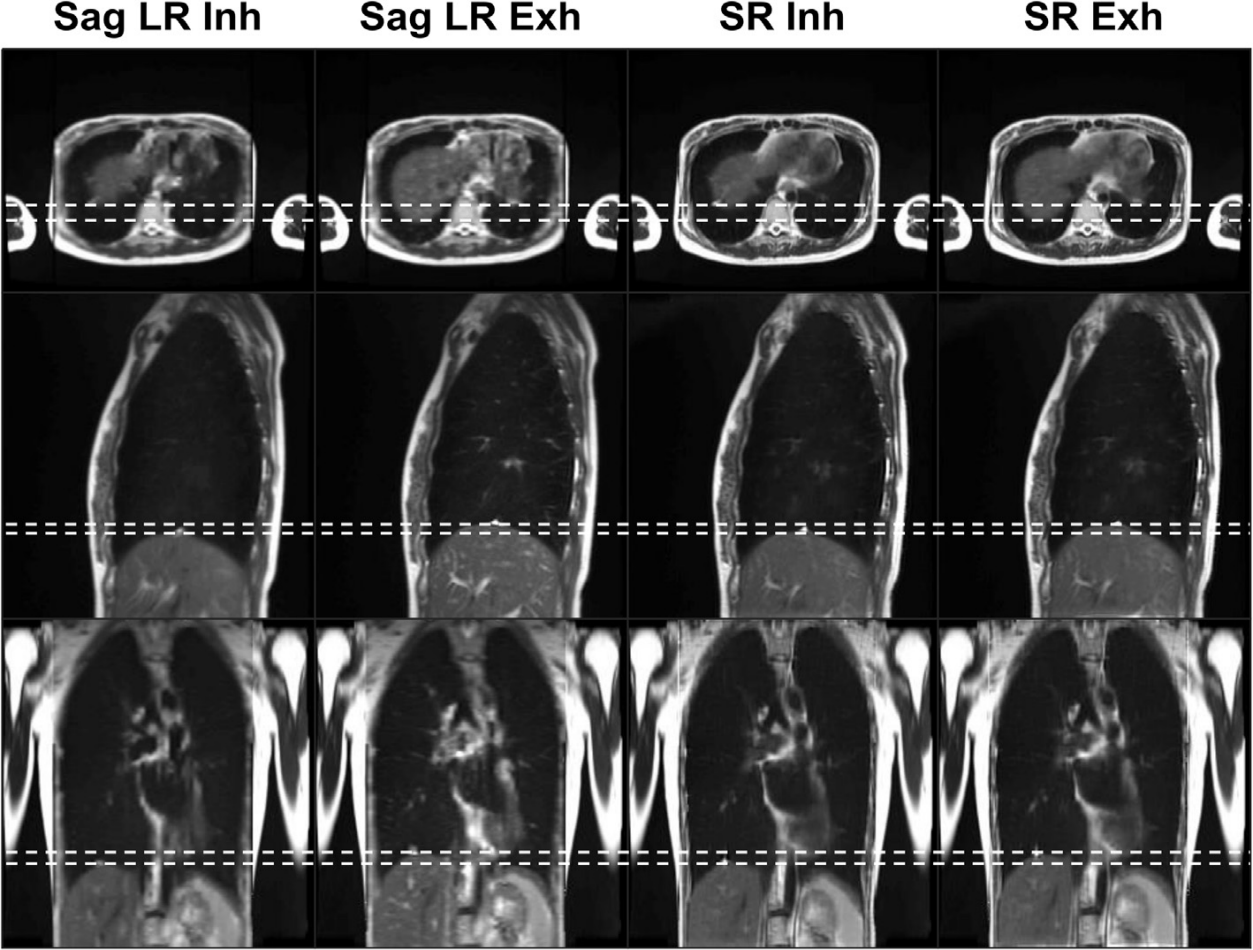
Comparison of the four-dimensional super-resolution (SR) reconstruction to the sagittal four-dimensional low-resolution (LR) reconstruction. Dashed white lines demarcate the displacement of the inhalation (Inh) and exhalation (Exh) phases.

For eight volunteers, distortion corrected super-resolution 4D/MidP-T2w MRI (1.0 × 1.0 × 1.0 mm^3^) were calculated using a fully automatic workflow. Low-resolution 4D-T2w MRI was reconstructed in 3.2 min for each orientation and total reconstruction time for super-resolution 4D/MidP-T2w was less than 23 min. Super-resolution 4D-T2w MRI exhibited no missing-data artefacts and, when compared to the initially binned low-resolution 4D-T2w MRI, displayed greatly reduced stitching artefacts; as demonstrated in the movie of Supplemental Digital Content 1 (15 dynamics). Fig. 2 shows an example comparing super-resolution MidP-T2w MRI, the initial guess for super-resolution reconstruction (mean of all 16 interpolated low-resolution copies) and the low-resolution MidP sagittal and axial images (averaged over 8 interpolated copies). Super-resolution reconstruction maintained the in-plane quality and image features of both the axial and sagittal orientations, whilst increasing the image sharpness in the coronal plane. The motion information exhibited by the low-resolution sagittal 4D-T2w MRI was well preserved in the super-resolution 4D-T2w MRI, as shown in Fig. 3.

The measured anterior boundary skin-air resolution in the central axial slice of the unprocessed ground-truth, initial guess and super-resolution reconstructed images was: (mean and standard deviation) 1.4 ± 0.2, 2.2 ± 0.2 and 1.5 ± 0.2 mm. The small resolution difference between the native and super-resolution images suggests that the super-resolution reconstruction can overcome the smoothing associated with the registration and interpolation steps.

**Fig. 4:**
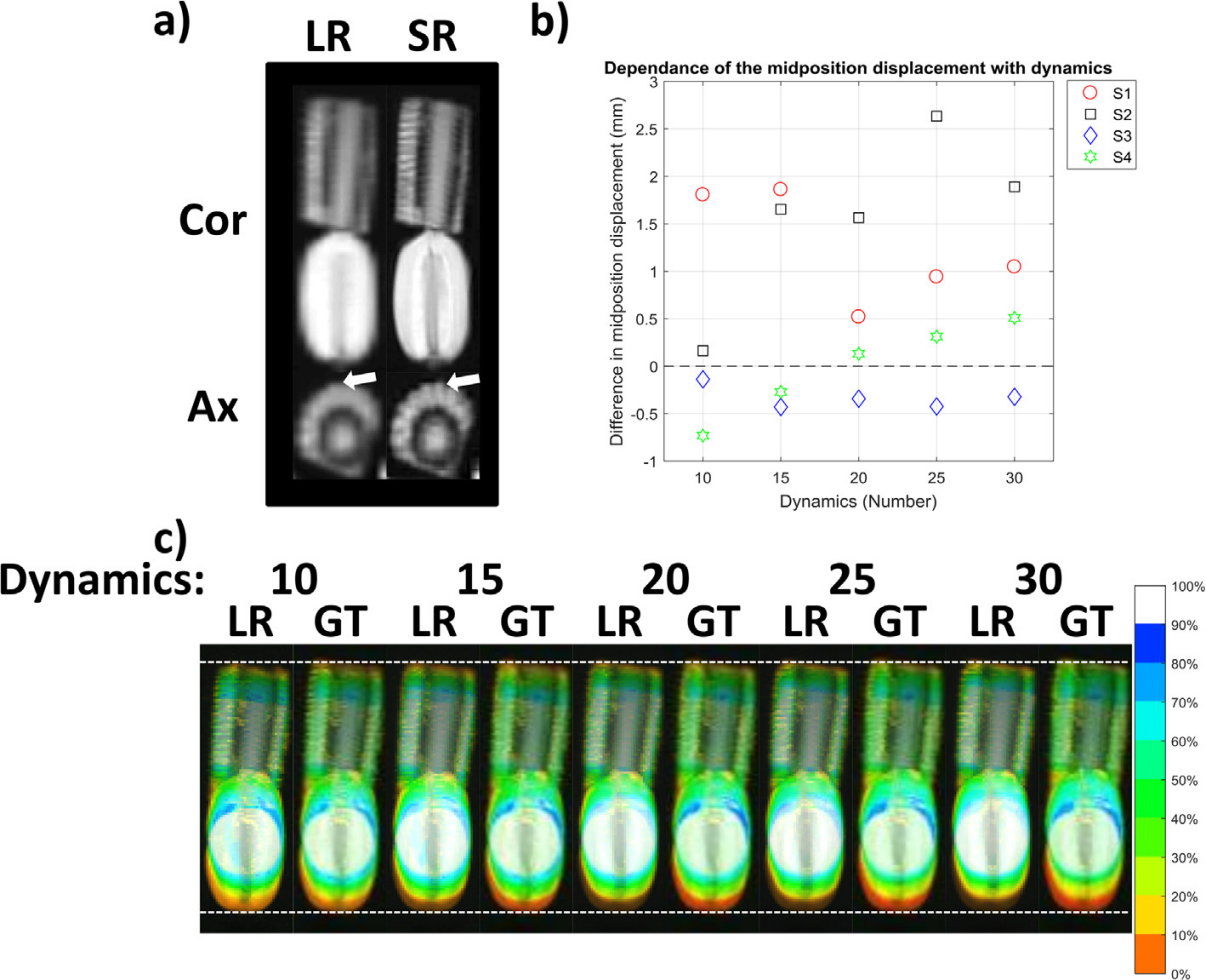
(a) Low-resolution (LR) and super-resolution (SR) images of a motion phantom (waveform S3), for one coronal (Cor) and axial (Ax) slice in midposition. Sweetcorn kernels (white arrow) are more easily distinguished in the SR images than in the LR images. (b) Depicts the difference in midposition displacement of the phantom versus the number of dynamics for the sinuosoidal (S1-S2) and volunteer (S3-S4) waveforms in the low-resolution T2w MRI (Motion-model) and in the ground-truth (GT) images. (c) LR and GT colour-intensity projection images of one coronal slice for different numbers of dynamics (waveform S4). The LR images show a slight underestimation of motion. Colour encodes time spent in each position. 100% of the time means that part of the phantom is always in the same position. Dashed white lines show exhalation and inhalation (top and bottom) for the GT data set of 30 dynamics.

As illustrated in the axial view presented in Fig. 4a, super-resolution reconstruction results in an increase in image sharpness and reduction of partial volume effects, better enabling visualisation of features such as the sweetcorn kernels within the test-object. As shown in Fig. 4b, the differences (mean and standard deviation over all dynamics) in the MidP displacement calculated from the low-resolution model and the unprocessed ground-truth images for the sinusoidal waveforms (S1-S2) were: 1.2 ± 0.6 mm and 1.6 ± 0.9 mm, and for the volunteer waveforms (S3-S4) were: −0.3 ± 0.1 mm and 0.0 ± 0.5 mm. The motion range in the 4D-T2w MRI slightly underestimated the full motion range of the unprocessed ground-truth images. This is reflected in Fig. 4c, where the CIPs of S4 reveal that this slight underestimation of motion occurred in inhalation.

**Fig. 5:**
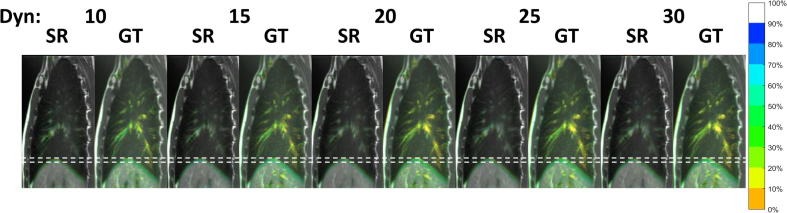
Colour-intensity projection images displaying time-weighted motion information contained in the dynamics (Dyn) of the unprocessed sagittal ground-truth (GT) images and respiratory phases of the super-resolution (SR) images of volunteer 1. Dashed white lines aid visual comparison of inhalation and exhalation. In the lung, colour approximately represents time spent in each position. 100% (grayscale) means that image intensity is not changing in that position for all respiratory phases. The respiratory motion in the GT images is preserved in the SR images, which can be recognised by comparing the extent of the liver dome motion. Pulsations due to cardiac motion in the GT images are reduced in the SR images. (For interpretation of the references to colour in this figure legend, the reader is referred to the web version of this article.)

CIPs were generated from the unprocessed ground-truth images and from the results of super-resolution reconstruction; an example is shown in Fig. 5. In terms of diaphragm motion, the CIPs from the super-resolution reconstruction were in good agreement when compared to those calculated from the unprocessed ground-truth images. The observable range of motion exhibited in CIPs only varied slightly with number of dynamics. Due to averaging, cardiovascular motion and pulsations observed in the unsorted data were reduced in the super-resolution reconstruction.

**Fig. 6:**
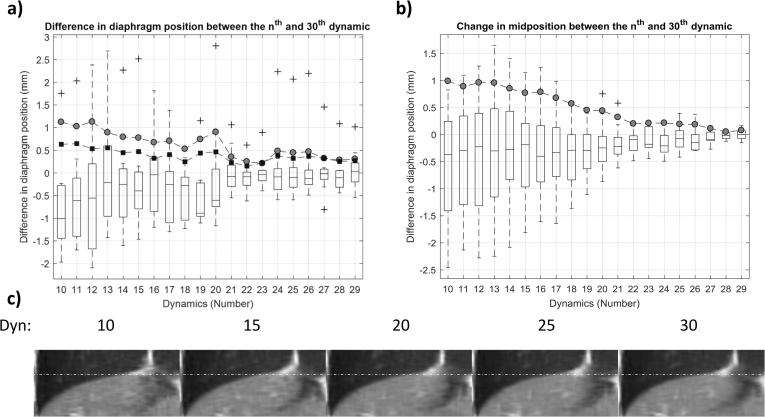
(a) Differences in diaphragm position between the low-resolution sagittal midposition images obtained from *N* (10 to 29) and 30 dynamics. Boxplots summarize the results from the eight volunteers. Mean absolute differences are depicted by grey circles (uncorrected) and black squares (corrected for drift in midposition). (b) Changes in midposition with number of dynamics; boxplots summarize results for different volunteers. The mean absolute difference in midposition compared to the full 30 dynamics is depicted by grey circles. (c) Coronal views of super-resolution midposition images of volunteer 1 for different number of dynamics (Dyn). Images exhibit high similarity. White dashed lines aid comparison of the diaphragm position.

As shown in Fig. 6a, a negative correlation (Pearson’s *r* = −0.89) was found between the absolute differences in diaphragm position of MidP calculated from *N* and 30 dynamics. The average absolute differences, displayed as grey circles, ranged from 1.1 ± 0.6 to 0.3 ± 0.3 mm (mean and standard deviation over volunteers) for 10 and 29 dynamics, respectively, and all were within 1.1 mm. As displayed in Fig. 6b, a drift in MidP was observed for all volunteers and the mean absolute difference between 10 and 30 dynamics was 1.0 ± 0.8 mm over an average acquisition of 5.2 min. The differences in diaphragm position were corrected for the change in MidP and the absolute differences (mean and standard deviation) were 0.6 ± 0.6 and 0.3 ± 0.4 mm for 10 and 29 dynamics (black squares in Fig. 6a). Fig. 6c shows super-resolution MidP images for varying number of dynamics. Similarity to the image obtained from 30 dynamics slightly increased with number of dynamics, but was nevertheless high for all images.

## Discussion

In this article, an automated binning and super-resolution reconstruction method was introduced to obtain high-resolution distortion corrected 4D/MidP-T2w MRI. Super-resolution reconstruction was enabled by combining copies of low-resolution axial and sagittal MidP-MRI, which were independently reconstructed for each raw image orientation. In phantom experiments, the MidP displacement exhibited by low-resolution 4D-T2w MRI was at most 2.6 mm different to that estimated from the unprocessed ground-truth images. Data subsampling experiments demonstrated that 10 dynamic acquisitions in each orientation were sufficient to obtain representative MidP-T2w MRI. Employing super-resolution reconstruction reduced the smoothing associated with the registration and interpolation steps in the workflow, facilitating visualisation of small structures with high T2 contrast. The resulting high quality 4D-T2w MRI exhibits thin slices whilst maintaining sufficient signal-to-noise ratio and FoV to facilitate RTP of anatomy undergoing respiratory motion. Furthermore, the suggested motion-model permits a reduction in stitching artefacts and mitigates missing-data artefacts by sorting images on a slice-by-slice basis; avoiding unnecessary long acquisitions.

For all waveforms in the phantom experiment, the MidP displacement in the low-resolution 4D-MRI exhibited a maximum error of 1.6 ± 0.9 mm compared to the ground-truth image data, suggesting that the MidP displacement was well preserved within the proposed workflow. The remaining differences might be associated with the reduction of stitching artefacts.

Compared to the unprocessed CIPs of the phantom and volunteer measurements, the low-resolution and super-resolution reconstruction CIPs both showed a slight underestimation towards inhalation. Overall, the range of diaphragmatic motion was well preserved in the super-resolution reconstruction CIPs; further suggesting that the motion-model sufficiently represents the motion range.

For all volunteer MidP images (obtained from *N* dynamics), the average diaphragm displacement was concurrent with that at 30 dynamics to within 1.1 mm, which was in rough agreement with the phantom results.

Our approach ensures data completeness by sorting images on a slice-by-slice basis. This enables faster acquisition compared to alternative binning strategies [19], [21]. Due to the exploitation of IRS, the proposed method is fully automatic; saving valuable time, for instance by avoiding the setup of a respiratory bellows [17]. In contrast to other methods [16], [19], a 3D gradient non-linearity distortion correction is applied to optimise geometrical fidelity for RTP. Additionally, cardiovascular flow and motion are suppressed by the super-resolution reconstruction, which facilitates RTP by reduction of obfuscating features. In comparison to a recently published method, in which 4D-T2w MRI is obtained by applying the motion information from a 4D-T1w MRI (radial stack-of-stars sequence) to 3D-T2w MRI [5], [42], 4D-T2w MRI was obtained with a higher resolution and required a considerably shorter time to reconstruct. The 3D radial stack-of-stars trajectory provides a large degree of flexibility regarding the imaging contrast, in particular a balanced implementation with a mixed T2/T1 contrast is possible and clinically feasible reconstruction times were demonstrated [15]. Likewise, the proposed super-resolution method could be applied to generate 4D-MRI of any contrast accessible using a fast 2D imaging sequence. Additionally, other 4D imaging contrasts could be acquired by transferring the motion information obtained during the motion-modelling workflow to other volumetric images [5]. Identifying which 4D imaging technique is most suited for characterising motion of treatment targets and organs at risk is an open question in lung RTP [3].

A limited number of healthy volunteers were scanned and might not be representative of lung cancer patients, since patients may exhibit less regular respiratory patterns due to compromised lung function. In this work a sagittal and axial acquisition strategy was employed to optimise image quality for contouring in RTP, which is commonly carried out in the axial plane. An axial and coronal acquisition strategy could be employed to exploit the higher acceleration factor in parallel imaging due to favourable orientation of coil elements compared to a sagittal acquisition. The high specific absorption rate (SAR) of the HASTE sequence may limit the applicability of the acquisition strategy at higher field strengths or for patients with implants. However, SAR can be decreased by reduction of the refocusing flip-angle or by increasing the repetition time. The current acquisition time of 16.8 ± 1.2 min (30 dynamics) is twice that of existing low-resolution methods [21], [33]. However, acquisition time could be competitive and viable for a hybrid MRIgRT treatment setup if only 10 dynamics were acquired, which we showed to be sufficient for MidP-T2w MRI. The reconstruction time of the non-optimised MATLAB prototype is currently too long for clinical use on MRIgRT systems, but has scope for improvement, e.g. by exploiting a multi-core cluster to accelerate calculation of the parallelisable components [15].

By using available tumour trajectory information to compensate for respiratory motion, the MidP RTP technique results in almost the same planning margin outcomes as idealised gated radiotherapy [8]. The presented 4D/MidP-T2w MRI could be employed alongside 4D/MidP-CT to facilitate target delineation and also to update tumour trajectory information and margins throughout treatment. In addition to MidP images, alternative planning images, e.g. midventilation [8], could be generated with the presented approach, since the whole respiratory cycle is covered.

We have demonstrated the feasibility of obtaining high-resolution 4D/MidP-T2w MRI (1.0 × 1.0 × 1.0 mm^3^) from axial and sagittal 2D-T2w MRI with a binning and super-resolution reconstruction method. Additionally, 10 repeated dynamic measurements of data were found sufficient to obtain representative MidP-T2w MRI. The resulting 4D/MidP-T2w MRI were distortion corrected, exhibited reduced stitching artefacts, and were free of data incompleteness artefacts. Super-resolution 4D/MidP-T2w MRI is a promising technique for hybrid MRIgRT systems and to facilitate target delineation for treatment planning.

## Conflict of interest

The Institute of Cancer Research and Royal Marsden NHS Foundation Trust is part of the Elekta MR Linac Research Consortium.

## Figures and Tables

**Supplementary Fig. 1:**
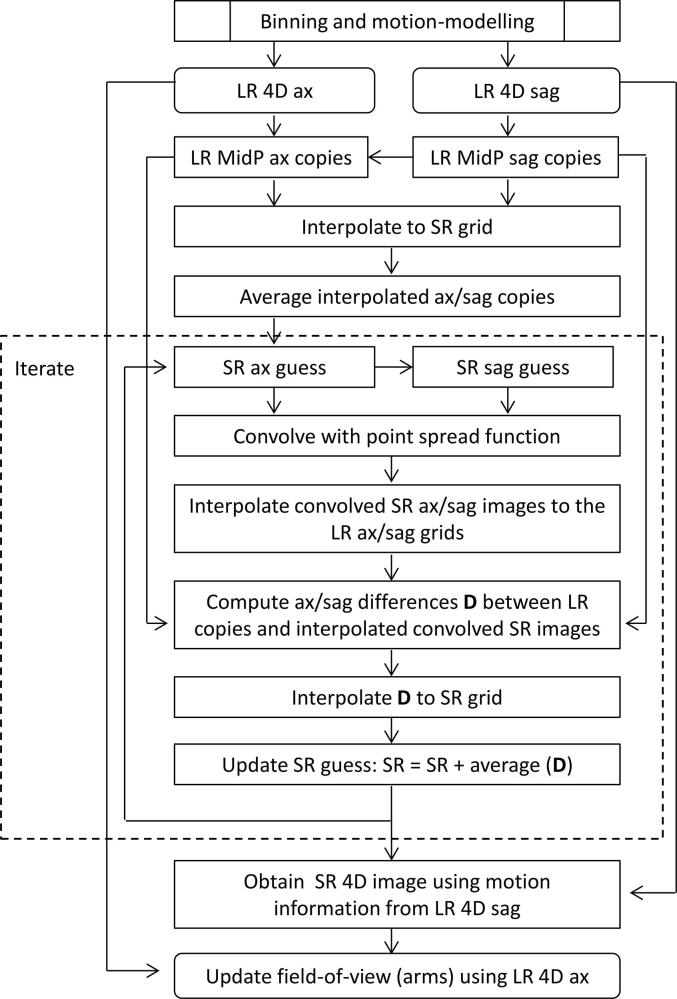


## Appendix A Supplementary data

Supplementary data 1

Supplemental Digital Content 1
