# Supplementary material for: Super-resolution T2-weighted 4D MRI for image guided radiotherapy
Source: Radiother Oncol. 2018 Dec;129(3):486–93. doi: 10.1016/j.radonc.2018.05.015 (PMC6294732; doi:10.1016/j.radonc.2018.05.015)
Supplement: Supplementary data 1 [file mmc1.docx]

**SUPPLEMENTAL MATERIAL**

**Super-resolution Reconstruction**

The super-resolution (SR) reconstruction restored high frequency information by iteratively back-projecting the differences between the low-resolution (LR) midposition (MidP) copies and a continuously updated SR image; which was achieved by minimising the objective function:

$$\underline{\mathrm{SR}}=argmin_{\underline{\mathrm{SR}}}\sum_{k=1}^{N} \left. \left( \hat{H}\hat{B}\underline{\mathrm{SR}}-{\underline{\mathrm{LR}}}_{k} \right) \right.^{2}$$

Where k = 1 to N, denotes the number of LR images. The operators $\hat{H}$ and $\hat{B}$ describe application of downsampling and blurring (non-isotropic Gaussian point spread function). Unlike typical SR objective functions [28], no geometrical operator is required since the images were registered prior to reconstruction. Details regarding the implementation of the reconstruction can be found in Supplemental Figure 1.


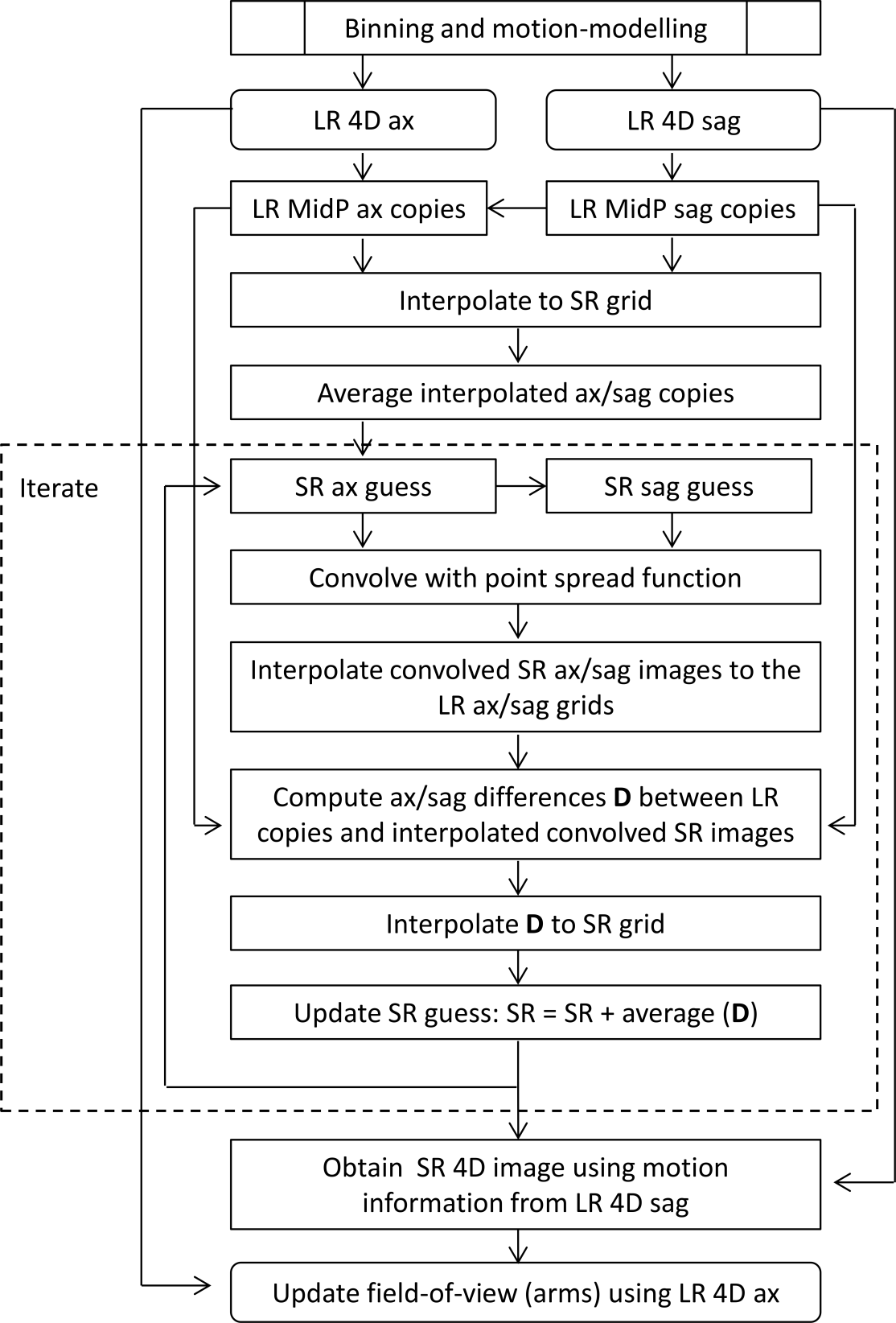


**Supplemental Figure 1**: Details of the super-resolution (SR) reconstruction workflow. The low-resolution (LR) midposition (MidP) axial (ax) copies were obtained by registering each phase of LR 4D ax to the LR MidP sagittal (sag) copies. The SR sag guess was obtained by reformatting the SR ax guess image. The size of the Gaussian point spread function was 1.8x1.8x5 mm^3^. Eight iterations were employed.
